# High expression of PDE4D correlates with poor prognosis and clinical progression in pancreaticductal adenocarcinoma

**DOI:** 10.7150/jca.35443

**Published:** 2019-10-17

**Authors:** Fude Liu, Jieyi Ma, Kebing Wang, Zhi Li, Qingping Jiang, Hui Chen, Wen Li, Jintang Xia

**Affiliations:** 1 Department of General Surgery, The Third Affiliated Hospital, Guangzhou Medical University, Guangzhou, Guangdong 510150, China; 2 Laboratory of General Surgery, The First Affiliated Hospital, Sun Yat-sen University, Guangzhou, Guangdong 510080, China; 3 Department of Pathology, The First Affiliated Hospital, Sun Yat-sen University, Guangzhou, Guangdong 510080, China; 4 Department of Pathology, The Third Affiliated Hospital, Guangzhou Medical University, Guangzhou, Guangdong 510150, China

**Keywords:** phosphodiesterase 4D, pancreatic ductal adenocarcinoma, prognosis, β-catenin

## Abstract

Background: Phosphodiesterase 4D (PDE4D) has recently been reported as an oncogene in various types of human cancers. However, the expression and significance of PDE4D in pancreatic ductal adenocarcinoma (PDAC) have not been elucidated.

**Methods**: Immunohistochemistry (IHC) was used to examine the expression of PDE4D in 104 clinicopathologically characterized PDAC cases. PDE4D expression in paired tumor tissues and adjacent noncancerous tissues were detected by western blotting and real time qRT-PCR. The correlation of PDE4D expression levels with clinicopathological features and prognosis in patients were analyzed by univariate and multivariate methods. Effect of PDE4D on pancreatic cancer cells was detected by cell migration and invasion assays.

**Results**: We found that PDE4D was significantly up-regulated in PDAC tumor tissues compared to those paired adjacent noncancerous tissues at both protein and mRNA levels. High level of PDE4D was significantly associated with clinical stage (*P* = 0.004), T classification (*P* = 0.003), lymph node metastasis (*P* = 0.022) and liver metastasis (*P* = 0.038). Patients with higher levels of PDE4D had shorter overall survival time contrast with those with lower PDE4D expression (*P* = 0.002). Multivariate analysis indicated that PDE4D may be an independent prognostic factor for PDAC. PDE4D depletion significantly suppressed β-catenin and Snail expression as well as the migration and invasion abilities of pancreatic cancer cells.

**Conclusions**: Our study reveals that PDE4D up-regulated in PDAC was closely associated with poor prognosis of PDAC patients and multiple aggressive clinicopathological characteristics. PDE4D could be a useful prognostic biomarker and therapeutic target for PDAC.

## Introduction

Pancreatic ductal adenocarcinoma (PDAC) is known as one of the most devastating cancer with an extremely poor prognosis. According to the report of GLOBOCAN 2012, there were 65,727 Chinese diagnosed with PDAC, among which 63,662 cases died, accounting for 96.86% of the newly diagnosed PDAC cases in China during 2012 1. PDAC is the fourth leading cause of cancer-related death in the United States and the seventh in China with a median survival of 3-6 months and a 5-year survival rate of less than 5% 2-4. Despite decades of effort to this disease, the survival rate remains no apparent improvement 5. So far, the only curative treatment available for PDAC is surgical resection. Unfortunately the vast majority of patients are not diagnosed until late stage in their disease, whereas less than 20% of patients are surgically respectable at the time of diagnosis 6, 7. Strong local invasion and early distant metastasis are the main causes for the worse prognosis of PDAC 8, 9.

Phosphodiesterases (PDEs) belong to metalloproteinase, which specially degrade the secondary messengers cAMP and cGMP 10, 11. According to their subcellular distributions, amino acid sequences and substrate-specificity, PDEs are grouped into 11 families (PDE1-11). PDE4D (cAMP specific 3', 5'-cyclic phosphodiesterase 4D) belongs to PDE4 families which contains at least 9 isoforms and all the 9 isoforms have the catalytic domain of phosphodiesterase in their carboxyl terminus. PDE4D plays a very important role in regulating cAMP hydrolyzation but does not affect cGMP 12, 13. More recently, PDE4D has been identified as a novel tumor-promoting molecular which represents a unique targetable enzyme in various human cancers, such as lung, prostate, melanoma, ovarian, endometrial, colorectal cancer and gastric cancers 14-17. However, the PDE4D expression and prognostic significance in PDAC has not been reported up to now.

In the present study, we found that the expression of PDE4D was increased in the PDAC samples, compared to their adjacent non-tumor tissues. High expression of PDE4D was correlated with poor prognosis and clinical progression in patients of PDAC. Furthermore, *in vitro* experiments indicated that PDE4D may promote migration and invasion abilities of PDAC cells through β-catenin and Snail.

## Material and Methods

### Patients and Tissue Specimens

The tissues, which were archived and formalin-fixed paraffin-embedded, were obtained from104 patients with diagnosis of PDAC who had undergone surgical resection or biopsy from September 2003 to March 2011 in the Department of Hepatobiliary Surgery, the First Affiliated Hospital of Sun Yat-sen University, China. There were 67 patients initially received radical resection and 37 patients received palliative operation. Ultrasound and computed tomography scans had been performed on all of the 104 patients prior to their operation. Postoperative chemotherapy was performed to 23 patients with advanced stage of PDAC, whereas no radiotherapy was performed to any of those patients.

To detect the mRNA and protein levels of PDE4D expression, four matched pairs of fresh PDAC tumor and adjacent non-tumor tissue samples, which at less 2 cm away from the tumor border, were also obtained from the pancreatectomy specimens. By using histopathology analysis with HE staining in frozen sections, all samples had been confirmed that the cancer lesions comprised of more than 70% cancer cells without necrosis, and adjacent noncancerous tissues did not have tumor cells. The informed consent of all the patients and the permission from Medical Ethical Committee of the First Affiliated Hospital, Sun Yat-sen University had been obtained before the use of clinical specimens in this study.

### RNA isolation and qRT-PCR

The total RNA which derived from tumor tissues of PDAC and the matched paired adjacent noncancerous tissues were extracted using the Trizol reagent (Invitrogen; Carlsbad, USA) according to the manufacturer's protocol. Total RNA was handled with RNAase-free DNase in advance, and 2 μg RNA from each specimen was used for cDNA synthesis. qRT-PCR was conducted with LightCycler® 480 SYBR Green Ⅰ Master on LightCycler480 instrument (Roche, Switzerland). Primer sequences used in this study are listed as the following: PDE4D, forward: 5'-ACCATTACCATGCTGATGTGGCCT-3' and reverse: 5'-ACACAGCCTCCAAAGCAGGTG -3'; GAPDH, forward: 5'-CTGACTTCAACAGCGACACC-3' and reverse: 5'-TGCTGTAGCCAAATTCGTTG-3'. GAPDH expression was used as an internal reference when conducting the data analysis.

### Western blotting

Samples were lysed in protein lysis buffer which was composed of 50 mM Tris (pH 7.5), 100 mM NaCl, 1 mM EDTA, 0.5% NP40, 0.5% TritonX-100, 2.5 mM sodium orthovanadate, 10 μM protease inhibitor cocktail, and 1 mM phenylmethylsulfonyl fluoride. Extracted protein was separated by 10% SDS-PAGE, and transferred onto PVDF membranes (Millipore, Bedford, USA). The membranes were blocked in 5% milk in 1 × TBST for one hour at room temperature, followed by incubation with the respective primary antibodies at 4°C overnight. After washed with TBST, the membranes were incubated with horseradish peroxidase conjugated secondary antibodies (1:5000; CST, USA). An enhanced chemiluminescence (ECL) kit (Millipore, Bedford, USA) was used to visualize protein bands on PVDF membranes. Primary antibodies used in this study were listed as the following: mouse anti-GAPDH antibody (1:3000, Kangcheng, Shanghai, China), rabbit anti-PDE4D antibody (1:1000, Proteintech, USA), mouse anti-β-Catenin antibody (1:1000, BD, USA), mouse anti-N-cadherin antibody and rabbit anti-Snail (1:1000, CST, USA).

### Immunohistochemistry

Using an ultrasensitive kit (MXB; Fuzhou, China), immunohistochemical staining for PDE4D was performed on formalin-fixed, paraffin-embedded sections (4 μm thick), which were dewaxed in xylene, rehydrated in decreasing gradient ethanol, and then rinsed in PBS followed by antigen retrieval at 100°C with high-pressure steam treatment in 10 mM citrate buffer (pH, 6.0). After treating with peroxidase blocking solution to block the endogenous peroxidase activity for 10 min and normal nonimmunone serum for 10 min to reduce nonspecific binding, the sections were incubated with rabbit anti-PDE4D antibody at 4°C overnight, washed and then incubated with biotin-conjugated second antibody at room temperature for 10 min. Then the sections were sequentially incubated with streptavidin-peroxidase conjugate for 10 min and developing with 3, 3'-diaminobenzidine (DAB) as a chromogen substrate. The nuclei were stained with hematoxylin of Mayer.

Evaluation of the immunohistochemical staining was performed by two researchers independently. The PDE4D staining level was based on the proportion of staining area (positive tumor cells) and the intensity of staining. The staining scores were evaluated as follows. Staining intensity: 0 (no staining), 1 (light yellow, weak staining), 2 (yellow brown, moderate staining), 3 (brown color, strong staining). Staining area: 0 (no positive tumor cells), 1 (<10% positive tumor cells), 2 (10% to 35% positive tumor cells), 3 (35% to 70% positive tumor cells), 4 (>70% positive tumor cells). The PDE4D expression levels were determined by the immunoreactivity staining index (SI) ,which was calculated by the staining intensity and staining area scores and was assigned values of 0, 1, 2, 3, 4, 6, 8, 9, or 12, as previously described^18^, ^19^. For statistical analysis, we classified the PDE4D expression levels as two groups according to the SI: high expression level group (SI ≥ 6) and low expression level group (SI ≤ 4).

### Cell migration and invasion assays

Cell migration and invasion assays were performed to test the effect of PDE4D on pancreatic cancer cells PANC1 and Bxpc3, using cell culture inserts with PET membrane (Corning, USA) and BD BioCoat™ Matrigel™ Invasion Chamber (8 µm pore size) (BD Bioscience , USA), respectively. 24 h after cells were transfected with PDE4D siRNAs (si-PDE4D-1 and si-PDE4D-2) or negative control siRNA (siNC), cell migration and invasion assays were performed according to the manufacturer's protocols and as previously described 20. Migration assay and invasion assay were carried out at 7 h and 12 h for PANC1 cells respectively, while 20 h and 36 h for Bxpc3 cells respectively, after cells were suspended in serum free medium and added to the upper chambers of inserts.

### Statistical analysis

Statistical analysis was performed using the SPSS 22.0 statistical software package (SPSS Inc.; Chicago, USA). The correlations between PDE4D expression and clinicopathologic characteristics in PDAC was carried out with Pearson's Chi-square (χ^2^) test, Fisher's exact test and Spearman's rank correlation. The overall survival (OS) was calculated from the diagnosis date to the death date or the last follow-up date if death did not occur. Survival curve was described using the Kaplan-Meier method and compared using log-rank test between the high and low PDE4D expression cases. Univariate and multivariate Cox regression analyses were carried out to analyze the prognostic significance of PDE4D and other clinicopathologic parameters. *P* <0.05 was considered statistically difference.

## Results

### Clinicopathologic characteristics of patients with PDAC

The patients of PDAC contain 65 males and 39 females with age ranging from 27 to 78 years (median age: 61.5 years). The histological differentiation and clinical stages were defined according to the pathological tumor-node-metastasis (pTNM) classification system, based on the American Joint Committee on Cancer (AJCC) 21. There were 7 cases with well-differentiation, 67 cases with moderate-differentiation and 30 cases with poor- differentiation. Similarly, 10 cases were classified as stage I, 46 cases as stage II, 28 cases as stage III, and 20 cases as stage IV. The correlations of PDE4D with clinicopathologic features in PDAC patients are listed in Table 1.

### Expression of PDE4D is up-regulated in PDAC

**Figure 1:**
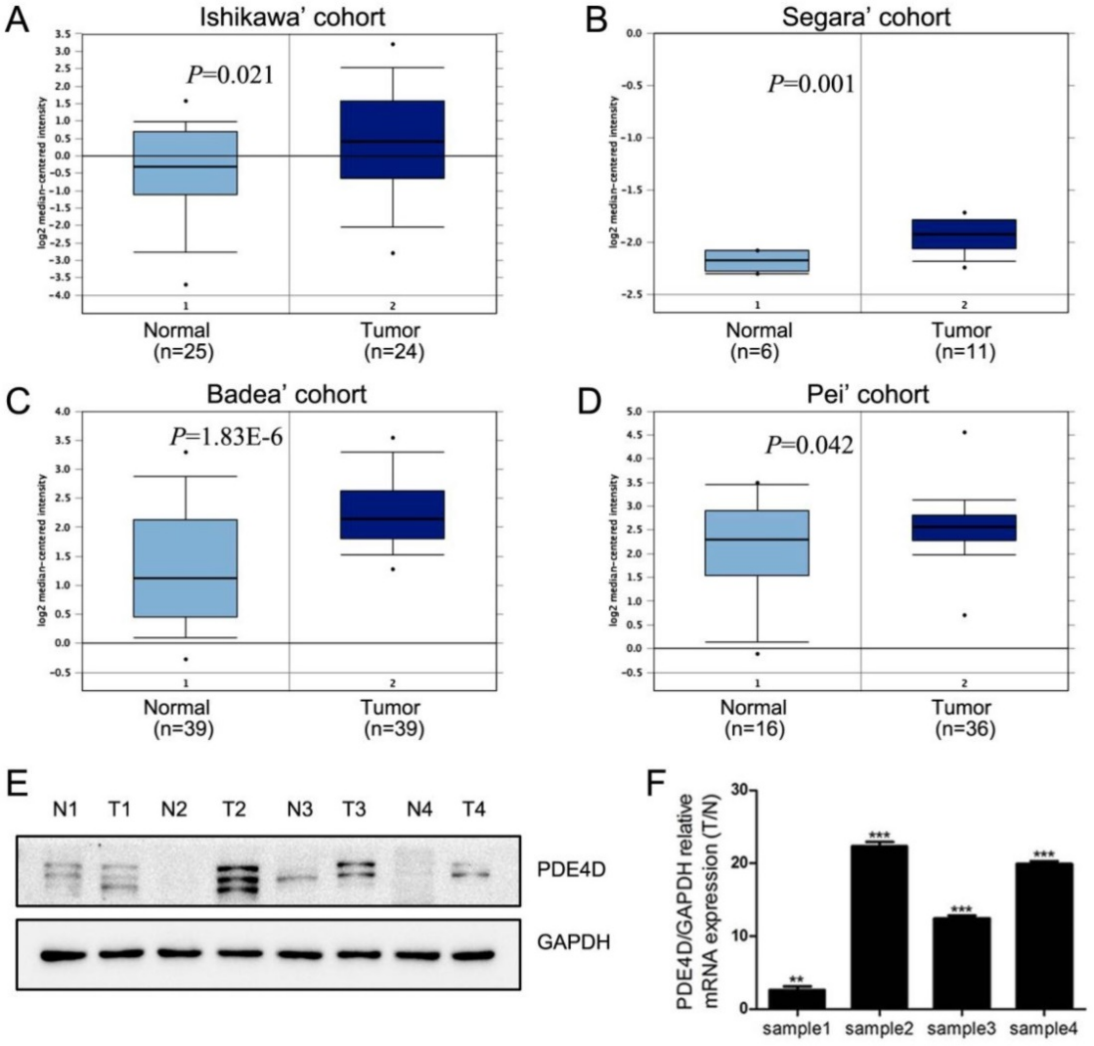
** The levels of PDE4D expression is elevated in PDAC. A-D)** Oncomine analysis of PDE4D expression in PDAC cohorts. **E)** Western blot showed protein levels of PDE4D expression in four PDAC tumor tissues (T) and their paired adjacent noncancerous tissues (N). **F)** qRT-PCR analysis showed the mRNA levels of PDE4D expression in tumor tissues compared to their paired adjacent noncancerous tissues (T/N). ** *P*< 0.01; *** *P*< 0.001.

We first used Oncomine database to analysis the expression of PDE4D in PDAC 22-25. These data indicated that PDE4D mRNA was significantly up-regulated in PDAC cohorts (Figure 1A-D). Similar trend was observed in our present study. The protein and mRNA levels of PDE4D were higher in all four PDAC lesion tissues (T), compared to their matched adjacent noncancerous tissues (N) as shown in the Figure 1E and 1F.

### Over expression of PDE4D is correlated with clinicopathologic features of PDAC

**Figure 2:**
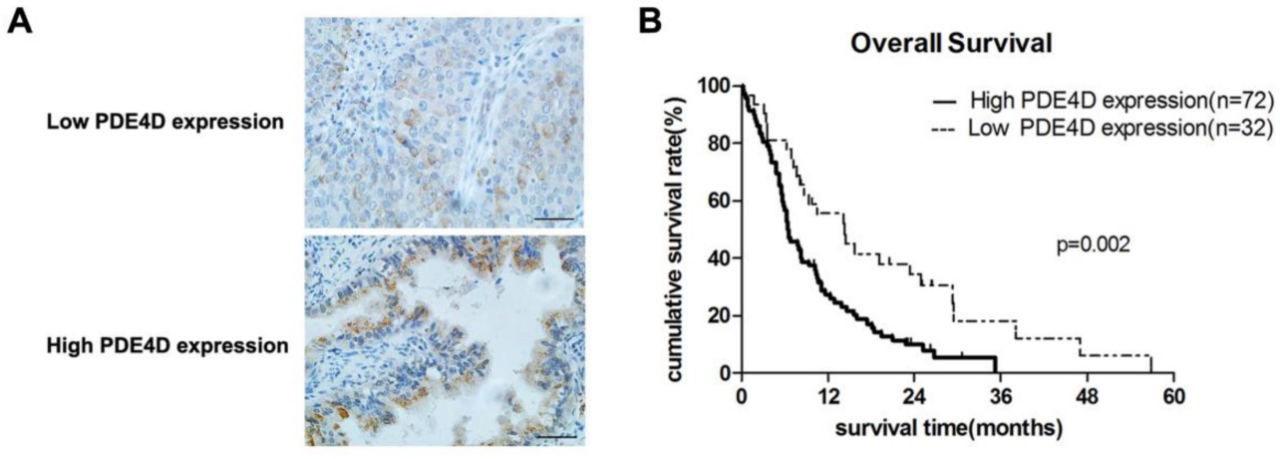
** The expression of PDE4D is associated with overall survival in patients with PDAC. A)** Immunohistochemical staining showed the low and high expression levels of PDE4D in PDAC tumor tissues, Scale bar: 100 μm. **B)** Kaplan-Meier survival curves show the significant statistical differences in survival times between the two groups. *P*-values were given by log-rank test.

Immunohistochemical staining was performed in 104 cases of PDAC. PDE4D was mainly localized in the cytoplasm of the PDAC tumor cells (Figure 2A). Analysis of the immunohistochemical staining in 104 cases of PDAC, we found that 72 cases (69.2%) have strong staining in lesion tissues with SI ≥6 which was classified as PDE4D high expression level group. The other 32 cases (30.8%) of PDAC have weak staining in lesion tissues with SI ≤4 which was classified as PDE4D low expression level group (Table 1).

The correlations between the protein expression of PDE4D and clinicopathologic features (including age, gender, clinical stage, differentiation and metastasis) were analyzed with Chi-square (χ2) test. The results showed that overexpression of PDE4D was significantly associated with clinical stage (*P* = 0.004), T classification (*P* = 0.003), N classification (*P* = 0.022), liver metastasis (*P* = 0.038) and tumor respectability (*P*=0.017). No obvious correlations were observed with age, gender, tumor location, size, histological differentiation and other clinicopathologic features (Table 1).

### The prognosis significance of PDE4D expression in PDAC patients

**Figure 3:**
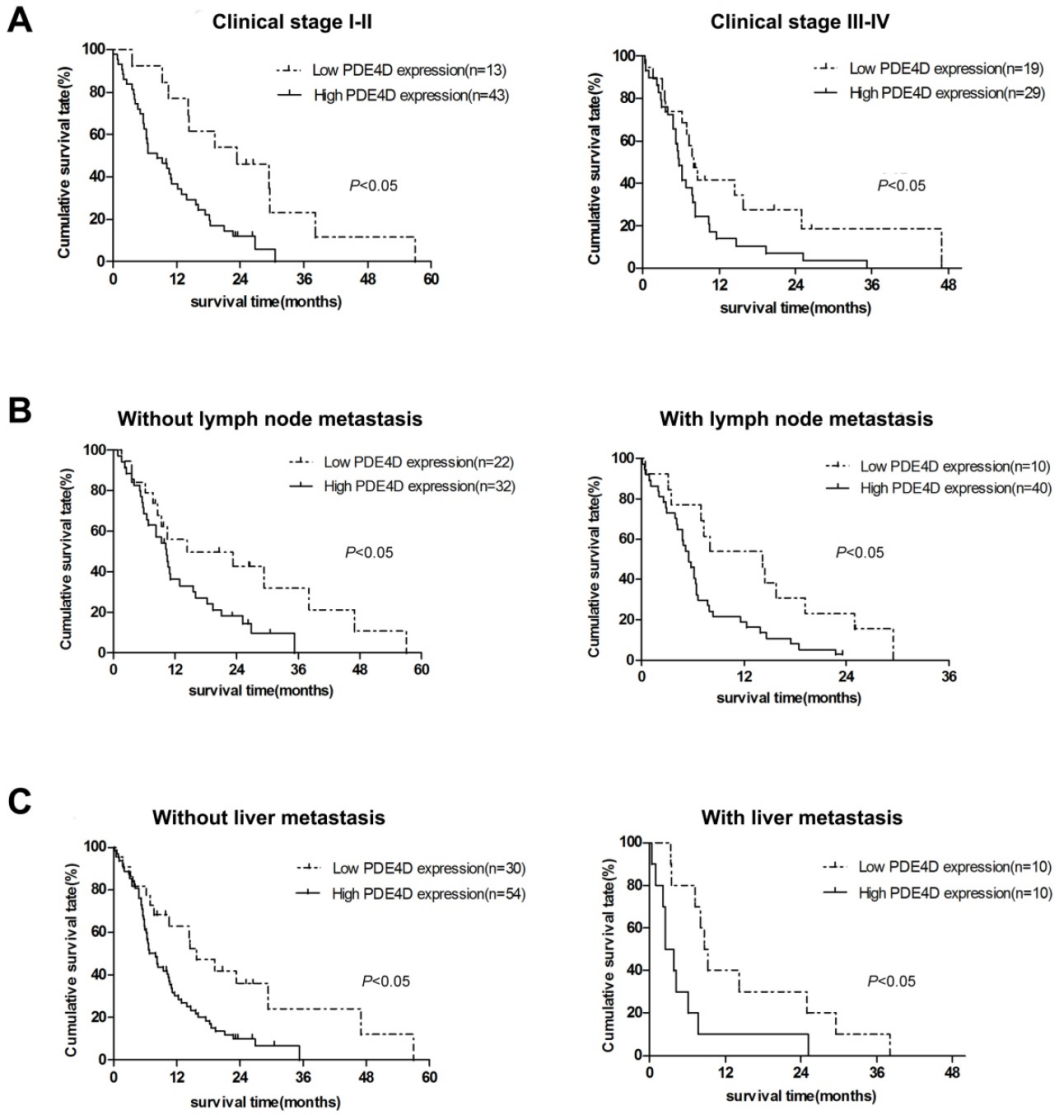
Kaplan-Meier survival curves indicate the significant differences in overall survival between PDAC patients with PDE4D-high and PDE4D-low according to clinical stage **(A),** lymph node metastasis status **(B)** and liver metastasis status** (C)**. *P*-values were given by log-rank test.

We examined PDE4D expression levels and the clinical follow-up information in all 104 PDAC patients by Kaplan-Meier analysis and log-rank test. Up to the end of follow-up time, 93 patients were died and 11 patients were still alive. Using univariate and multivariate analyses, the crude and adjusted relative risks of all cause of death in the 104 patients were assessed (Table 2). The median survival times of patients with high PDE4D expression level group was 6.5 months [95% confidence interval (CI): 7.7-11.4 months], which was significantly shorter than 14.3 months [95% CI: 11.5-21.2 months] for patients with low PDE4D expression level group (*P* = 0.002, log-rank text, Figure 2B). The results indicated that patients with high PDE4D expression levels have a worse prognosis compared to those with low PDE4D expression levels. Furthermore, we also examined the median survival times of the patients with different clinical stages, with or without lymph node and liver metastasis by Kaplan-Meier analyses. Our results showed that patients in PDE4D high expression level group with both early (I-II) and advanced clinical stage (III-IV) had markedly shorter survival times compared with those in PDE4D low expression level group (both *P* < 0.05; Figure 3A). Similar results were determined between patients in the following two expression groups, with and without lymph node metastasis (both *P* < 0.05; Figure 3B), with and without liver metastases (both *P* < 0.05; Figure 3C).

To determine whether PDE4D expression level is an independent prognostic factor, univariate and multivariate analyses were performed in the 104 cases of PDAC. As is shown in Table 2, univariate analysis suggested that PDE4D expression, tumor size, T classification, N classification and clinical stage were significant prognostic factors in PDAC. And multivariate analysis indicated that PDE4D expression, T classification and N classification were determined as independent prognostic factors for poor overall survival of PDAC patients.

### PDE4D regulates pancreatic cancer cells metastasis ability through β-catenin and Snail

**Figure 4:**
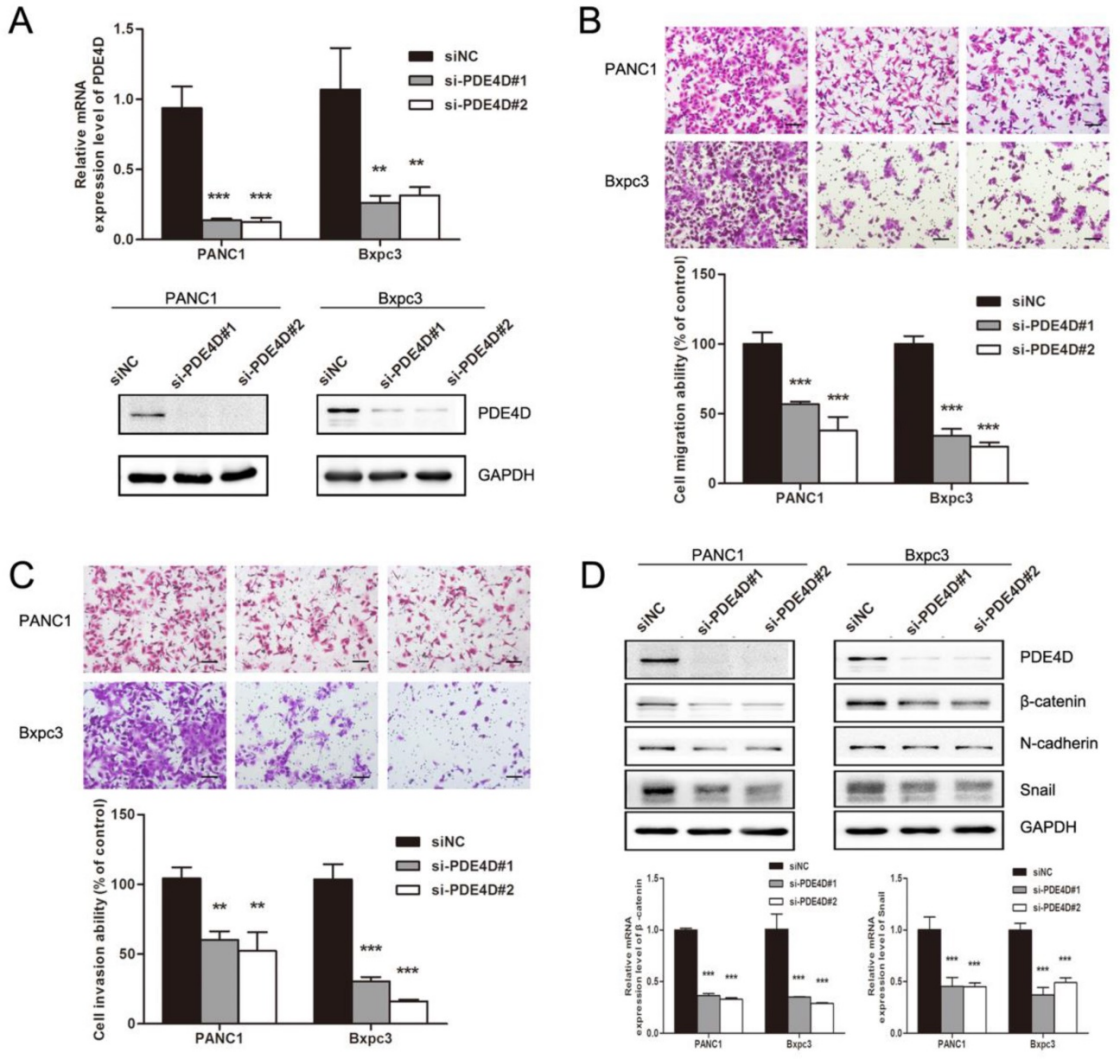
** Down-regulation of PDE4D suppressed the metastasis abilities of pancreatic cancer cells. A)** PDE4D siRNAs and siNC were transfected into pancreatic cancer cells PANC1 and Bxpc3. Western blot and qRT-PCR assay were performed 48h after transfection to examine the effect of siRNAs. **B-C)** Down-regulation of PDE4D significantly suppressed the migration and invasion abilities of pancreatic cancer cells. Scale bar: 200 μm. **D)** Western blotting and qPCR showed that the expression of β-catenin and Snail were down-regulated coincident with the suppression of PDE4D. Data are presented as mean ± SD. Statistical analysis was performed using Graph Pad Prism 5 with one-way ANOVA followed by Dunnett's multiple comparison test. **: *P*<0.01, ***: *P*<0.001.

In order to illuminate the effect of PDE4D on pancreatic cancer cells, cell migration and invasion assays were performed. As showed in Figure 4B and 4C, down-regulation of PDE4D with PDE4D siRNA significantly inhibited the migration and invasion abilities of pancreatic cancer cells indicating that PDE4D played an important role in pancreatic cancer cell metastasis. Mechanism by which PDE4D affects migration and invasion function of pancreatic cancer cells was further investigated. We found that β-catenin, Snail and N-cadherin protein levels were dramatically decreased in PDE4D silenced cells (Figure 4D). β-catenin is known as activator of the TCF/LEF family, leading to activation of Wnt signaling responsive genes and facilitate metastasis^26^. These data suggest that PDE4D over-expression might be a positive regulator of metastasis in pancreatic cancer cell though β-catenin/Snail pathway.

## Discussion

The poor outcome of PDAC attributes to early and aggressive local invasion, high recurrence rate, and poor sensitive to chemotherapy and radiotherapy 27, 28. Negative margin status and absence of lymph node metastases are critical for achieving a better survival rate of this devastating disease 29, 30.

cAMP functions as a second messenger in cell signaling transduction and cAMP signaling is regulated mainly by PDE family, especially PDE4D. PDE4D has recently been implicated as an oncogene and is correlated with the growth, proliferation and survival of cancer cells 14-16. High expression of PDE4D (subtype PDE4D2) significantly enhanced the proliferation of A375 melanoma cells and HGC-27 gastric cancer cells both *in vitro* and* in vivo*. Depletion of PDE4D with small interfering RNA (siRNA) caused apoptosis and growth retardation in various types of cancer cells, such as melanoma, breast, ovarian, endometrial, lung, colorectal and gastric cancers^14^, ^17^. Pharmacologic inhibition of PDE4D using small-molecule inhibitors induce tumor growth inhibition correlated to the SHH pathway in prostate cancer 31. Recent study suggested that the mitochondrial-associated apoptosis pathway was activated in shPDE4D-infected cells and the proapoptotic protein BIM was markedly elevated upon PDE4D depletion in both A549 and MB-231 cells 14. Our previous study found that down-regulation of PDE4D caused BIM-mediated cell growth arrest in colorectal cancer cells 17.

Elevated expression level of PDE4D has been reported to be associated with the proliferation, survival and prognostic in various cancers. However, seldom studies have reported the association of PDE4D with migration, invasion and cancer metastasis. In the present work, we found that PDE4D was up-regulated in the tumor tissue of PDAC at both mRNA and protein level compared with non-tumor tissues. For the first time, we demonstrated that overexpression of PDE4D was correlated with clinical stage (pTNM), lymph node metastases and liver metastases in PDAC. Besides, we showed that the group of patients with PDE4D high expression had a markedly shorter survival times compared to the group with PDE4D low expression in PDAC patients. Multivariate analysis of Cox regression indicated that overexpression of PDE4D was an independent factor of poor prognosis. Thus, PDE4D had potential for being used as a prognostic indicator biomarker in patients with PDAC.

Previous study found that PDE4D contributed to TGF-β1 stimulated-epithelial mesenchymal transition (EMT) in A549 cells 32. Recent study showed that PDE4D5 could promote BRAF-mutated melanoma cells invasion by interacting with FAK 33. Migration suppression was also found in PED4D knockdown prostate cancer cells 16. Increased levels of cAMP, the PDE4D target, specifically hindered PDAC cell motility through F-actin remodeling 34. Our* in vitro* study found that PDE4D could affect migration and invasion function of PDAC cell lines through β-catenin/Snail pathway.

β-catenin is a key component of Wnt signaling which is associated with cancer progression and maintains cancer stem cells properties. Studies have confirmed that β-catenin is critical in cancer cells invasion. Inhibition of AKT/GSK-3β/β-cateninpathway inhibited cell migration and invasion in hepatocellular carcinoma cells 35. Via regulating miR-182 and MMP9, β-catenin also altered cell invasion ability in HCT116 human colorectal carcinoma cells 36. In the present study, PDE4D silencing attenuated migration and invasion abilities of PDAC cells and decreased β-catenin level simultaneously. Previous studies have reported that knockdown of PDE4D resulted in EGFR/PI3K/AKT signaling inactivation in nasopharyngeal carcinoma cells 37. And EGF/AKT could contribute to phosphorylation of β-catenin and increased its protein level in cancer cells, therefore, enhanced β-catenin downstream transcriptional activity 38, 39. Our results indicated that PDE4D could regulate β-catenin and snail to promote cell invasion in PDAC cells. Whether this process is mediated by EGF/AKT signaling needs further investigation.

In summary, this study demonstrated that high expression of PDE4D was correlated with poor survival in PDAC. PDE4D depletion suppressed migration and invasion of PDAC cells through β-catenin/Snail pathway. Our study suggests that PDE4D can be used as a novel prognostic biomarker and a potential molecular therapeutic target in patients with PDAC.

## Figures and Tables

**Table 1:** Correlations between PDE4D expression and clinicopathologic features in PDAC

Clinicopathological feature	Number	Expression of PDE4D		P value(χ2 test)
		Low (n=32, 30.8%)	High (n=72, 69.2%)	
Age (years)				
≤60	45	14(31.1%)	31(68.9%)	0.947
>60	59	18(30.5%)	41(69.5%)	
Gender				
Male	65	23(35.4%)	42(64.6%)	0.188
female	39	9 (23.1%)	30(76.9%)	
Tumor location				
Head	83	28(33.7%)	55(66.3%)	0.193
Body/Tail	21	4 (19.0%)	17(81.0%)	
Size				
≤2cm	15	6 (40.0%)	9 (60.0%)	0.402
>2cm	89	26(29.2%)	63(70.8%)	
Clinical stage (pTNM)				
I	10	3 (30.0%)	7 (70.0%)	0.004*
II	46	10(21.7%)	36(78.3%)	
III	28	16(57.1%)	12(42.9%)	
IV	20	3 (15.0%)	17(85.0%)	
Histological differentiation				
Well	7	0 (0)	7 (100%)	0.161
Moderate/poor	97	32(33.0%)	65(67.0%)	
T classification				
T1	5	2 (40.0%)	3 (60.0%)	0.003*
T2	18	5 (27.8%)	13(72.2%)	
T3	44	6 (13.6%)	38(86.4%)	
T4	37	19(51.4%)	18(48.6%)	
N classification				
Absent	54	22(40.7%)	32(59.3%)	0.022*
Present	50	10(20.0%)	40(80.0%)	
Liver Metastasis				
Absent	84	22(26.2%)	62(73.8%)	0.038*
present	20	10(50.0%)	10(50.0%)	
Resectability				
Radical resection	67	26(38.8%)	41(61.2%)	0.017*
Palliative resection	37	6 (16.2%)	31(83.8%)	
Vital status				
Dead	93	26(28.0%)	67(72%)	0.071
Alive	11	6 (54.5%)	5 (45.5%)	

**Table 2:** Univariate and multivariate analyses of prognostic parameters for survival in patients with pancreatic ductal adenocarcinoma (PDAC)

Prognostic parameter	Univariate analysis				Multivariate analysis			
	RR	95%Cl		*P* value	RR	95%Cl		*P* value
		Lower	Upper			Lower	Upper	
Expression of PDE4D	2.099	1.295	3.4	0.003*	3.272	1.84	5.817	0.000*
Gender	1.056	0.691	1.614	0.802	0.961	0.601	1.538	0.868
Age	1.42	0.928	2.173	0.106	1.415	0.904	2.215	0.129
Size	2.055	1.112	3.795	0.021*	1.606	0.825	3.125	0.164
Tumor location	1.085	0.639	1.841	0.764	0.958	0.535	1.716	0.886
Pathologic differentiation	1.291	0.823	2.026	0.267	1.188	0.723	1.951	0.497
T classification	1.453	1.119	1.887	0.005*	1.715	1.227	2.395	0.002*
N classification	2.015	1.318	3.08	0.001*	1.717	1.092	2.702	0.019*
Liver metastasis	1.385	0.84	2.283	0.201	1.813	0.962	3.419	0.066
Clinical stage	1.358	1.09	1.691	0.006*	1.221	0.925	1.61	0.158

Univariate analysis and multivariate analyses were analyzed by Cox regression. *Statistically significant; RR: Relative Risk; 95% CI: 95% confidence interval.
